# Simulating a Measurement of the 2nd Knee in the Cosmic Ray Spectrum with an Atmospheric Fluorescence Telescope Tower Array

**DOI:** 10.1155/2014/278968

**Published:** 2014-03-09

**Authors:** Jiali Liu, Qunyu Yang, Yunxiang Bai, Zhen Cao

**Affiliations:** ^1^Physics Department, Kunming University, Kunming, Yunnan 650214, China; ^2^Physics Department, Yunnan University, Kunming, Yunnan 650214, China; ^3^Astro-Particle Physics Center, Institute of High Energy Physics, CAS, Beijing 100049, China

## Abstract

A fluorescence telescope tower array has been designed to measure cosmic rays in the energy range of 10^17^–10^18^ eV. A full Monte Carlo simulation, including air shower production, light generation and propagation, detector response, electronics, and trigger system, has been developed for that purpose. Using such a simulation tool, the detector configuration, which includes one main tower array and two side-trigger arrays, 24 telescopes in total, has been optimized. The aperture and the event rate have been estimated. Furthermore, the performance of the *X*_max⁡_ technique in measuring composition has also been studied.

## 1. Introduction

**Figure 1:**
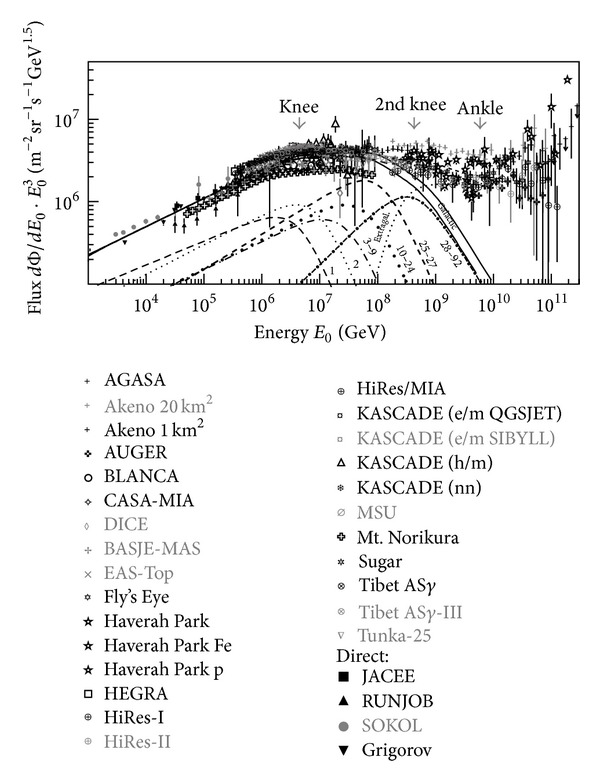
The all-particle cosmic ray energy spectrum multiplied by *E* ^3^. The dots represent the results observed by all experiments. The curves show the results calculated following the polygonato model [15]. The contribution of each single element (marked with different number *Z*) and the all-particle spectrum are shown separately.

The cosmic ray (CR) all-particle spectrum roughly follows a power law, that is, *dN*/*dE* ∝ *E* ^*γ*^. The index *γ* is not the same in different energy regions. It reveals different features as shown in Figure 1, where the flux is multiplied by *E* ^3^ [1]. One important feature is the knee at *E* = (3.2 ± 1.2) × 10^15^ eV, which the spectrum index *γ* changes from *γ* = −2.7 in the low energy region to *γ* = −3.1. The second important feature is the second knee around *E* = 4.0 × 10^17^ eV, where the spectrum index suddenly changes to *γ* = −3.3. Another important feature is the ankle at about *E* = 4.0 × 10^18^ eV, where the spectrum seems to become flat with *γ* = −2.7 again. The other important feature is Greisen-Zatsepin-Kuzmin suppression (called the GZK cutoff) [2] above about 6.0 × 10^19^ eV, which was observed for the first time by the HiRes experiment [3].

**Figure 2:**
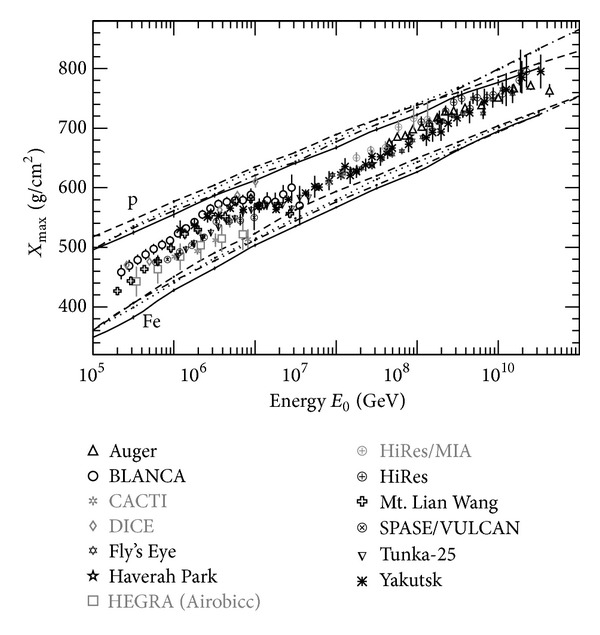
Mean *X*_max⁡_ distribution as a function of energy [16]. The dots are experimental results. The lines are simulation results for proton and iron predicted by Corsika [17] with the hadronic interaction model QGSJET 01 (solid line), QGSJET II-03 (dashed line), SIBYLL (dot line), and EPOS 1.6 (dot-dashed line) [18, 19].

Measuring these structures precisely will improve our understanding of the origin of CRs. However, because the dynamic range of individual experiments is limited and has its own energy scale and uncertainty, the observed results are not consistent among them. Furthermore, in composition observations, different experiments have different systematic uncertainties since they use different techniques, leading to the inconsistencies among the results as summarized in Figure 2. Therefore, to accurately measure the structures in the FF energy spectrum and the composition of CRs, it is ideal to have experiments being carried out at the same place with sufficient overlap among them. This would allow us to obtain a complete and self-consistent observation over the whole energy range from 100 TeV to several EeV.

The project of the Wide Field of View (FOV) Cherenkov/Fluorescence Telescope Array (WFCTA), composed of 24 telescopes, has been designed to reach these goals. One of the physical aims is to observe the energy spectrum and composition of CRs from 5.0 × 10^13^ eV to 2.0 × 10^18^ eV, covering both knees [4, 5]. To cover such a wide dynamic range, three observational stages with the same telescopes will be used. The first two stages will use the Cherenkov technique under two different configurations, covering the energy range of 5.0 × 10^13^ eV–1.0 × 10^16^ eV and 1.0 × 10^16^ eV–1.0 × 10^17^ eV. The first stage will overlap at lower energies with space/balloon borne measurements, which will provide a bridge between space/balloon borne measurements and ground based (low altitude) measurements. The third stage will focus on the energy range of 1.0 × 10^17^ eV–2.0 × 10^18^ eV, using the fluorescence technique by reconfigurating the telescopes. The overlap between these three stages will transfer the accurate energy scale of space/balloon borne experiments to the ground observatory and provide cross-calibration in energy. The focus of this paper is to estimate the performance of the fluorescence telescope tower array (the third stage of WFCTA) for the measurement of spectrum and composition around the second knee.  Section 2 describes the detector features and the telescope array configuration.  Section 3 will give details of the simulation chain. The simulation results on aperture, event rate, and on the performance of composition measurement are presented in Section 4.  Section 5 provides summary of results.

## 2. Detectors

**Figure 3:**
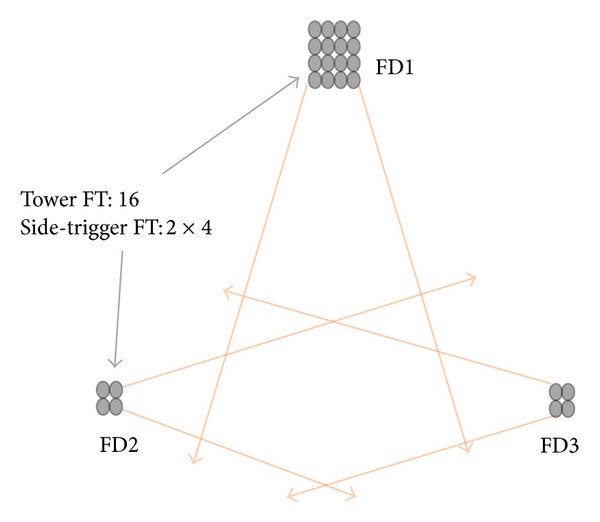
The configuration of the fluorescence telescope array. The solid circles are the fluorescence telescopes. The lines illustrate the detector FOV along the azimuth.

The proposed fluorescence detector array is composed of 24 telescopes, located at the three vertices of a triangle. As suggested by the configuration optimization, the chosen base line is 8 km and the height is 5 km. The three positions are denoted as FD1, FD2, and FD3, respectively. FD1 is configured as a 4 × 4 array, covering a range of 64° in azimuth and 56° in elevation starting from 3°. FD2 and FD3 are located at the bottom vertices, which are configured as a 2 × 2 array, covering a range of 32° in azimuth and 28° in elevation starting from 3°. Such a configuration is shown in Figure 3.

A 5.0 m^2^ light collecting mirror, composed of a 20 hexagonal mirror segments, with a reflectivity of 82% is used for each telescope. The image camera is made of 16 × 16 pixels. Each pixel is a 40 mm hexagonal photomultiplier tube (PMT) that has about a 1° × 1° FOV. Each PMT is read out by a 50 MHz flash ADC to provide a measurement of the shower waveform signals. A peak finding algorithm has been developed in order to provide an individual channel trigger using a field programmable gate array (FPGA). Three trigger levels are required for each event. The first level trigger is set by requiring the signal-noise ratio to be greater than 3.5*σ* for the FD1 array and 3*σ* for FD2 and FD3, where *σ* is the standard deviation of the total photoelectron noise within a running window of 320 ns. The second level trigger uses adjacent triggered tubes within a 6 × 6 box running over a telescope camera to form pattern recognition. The second level trigger is referred to as the telescope trigger. The “track-type” pattern requires at least six triggered pixels forming a straight line. The third level trigger is the event trigger which requires that both the tower array and one side array be triggered. The detailed description of the telescope can be found in [6].

## 3. Monte Carlo Simulation

In the simulation, cosmic rays are sampled isotropically and uniformly. The impact parameter, *R*_*p*_ (i.e., the distance from FD1 to the shower axis) is limited and is less than 10 km. An *E* ^−3^ spectrum is assumed for the resolution study. 728,555 proton and 854,342 iron primary showers have been generated in the energy range from 4 × 10^16^ eV to 2 × 10^18^ eV. To maintain statistics at high energies, 386,080 proton showers following an *E* ^−1^ spectrum covering the same energy range used in the resolution study are generated for the detector aperture estimation.

### 3.1. Air Shower Simulation

Given the shower energy and geometry (i.e., zenith angle, azimuth angle, *R*_*p*_, and core location), the shower longitudinal development is parameterized by a function with three parameters [7]: the maximum position of shower development *X*_max⁡_, the maximum number of charged particles *N*_max⁡_, and the width of the shower *σ*_*s*_, including their energy dependence, fluctuations, and the correlations between them. The number of charged particles *N*_ch_(*x*) at atmospheric depth *x* is calculated as

(1) $$\begin{array}{c} N_{\text{ch}}\left(x\right)=N_{\max}\exp\left\{-\frac{2{\left(x-X_{\max}\right)}^{2}}{\sigma_{s}^{2}{\left(x+2X_{\max}\right)}^{2}}\right\}. \end{array}$$

The shower lateral spread is taken into account in the simulation of photon production.

### 3.2. Photon Production and Light Propagation

The fluorescence and Cherenkov photons production are considered in detail as explained below.

Ultraviolet fluorescence light is generated as charged shower particles pass through the atmosphere. The fluorescence yield measured in [8] is used. Laterally, fluorescence photons are spread out using the Nishimura-Kamata-Greisen (NKG) function [9]

(2) $$\begin{array}{c} \rho \left(r\right)=\frac{N}{{\left(r_{0}\right)}^{2}}f\left(s,\frac{r}{r_{0}}\right), \end{array}$$

where *r*_0_ is the Moliere radius and *s* is the age of the shower. The normalized function *f* reads as

(3) f(s,rr0)=(rr0)s−2(1+rr0)s−4.5×Γ(4.5−s)[2πΓ(s)Γ(4.5−2s)].

**Figure 4:**
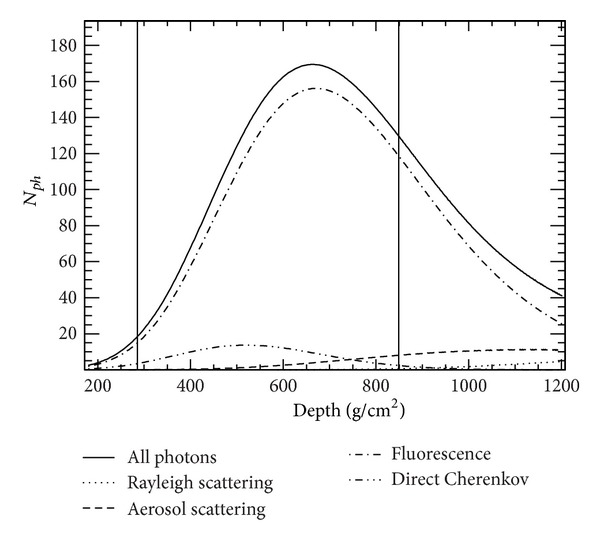
Profiles of all photons along with the shower longitudinal development that were produced by a shower in simulation. The solid curve represents the sum of all photons. The short-dot-dashed curve represents fluorescence photons. The long-dot-dashed curve represents direct Cherenkov components. The dashed curve represents Cherenkov photons scattered by aerosols. The lowest dotted curve represents Cherenkov photons scattered by atmospheric molecules (Rayleigh scattering). The range between two vertical lines is covered by FD1.

Cherenkov photons are also generated by charged particles if the particle energy is higher than the threshold energy [10]. Scattered by the atmospheric molecules (Rayleigh scattering) and aerosols (Mie scattering), photons are distributed in all directions according to corresponding phase functions. A standard desert aerosol model [11] with a scale height of 1 km and a horizontal attenuation length of 25 km is assumed in the simulation. The same model is used for estimating the photon flux attenuation due to scattering. A ray-tracing procedure is carried out to trace each photon to the photocathodes of PMTs once the photon arrives to the entrance of a telescope. All detector responses are considered in the ray-tracing procedure. In Figure 4, a typical example of profiles for all kinds of photons that are produced by a shower in the simulation is plotted as a function of slant atmospheric depth along with the shower longitudinal development, the range between two lines being covered by FD1. The detailed description of photon production and propagation can be found in [12, 13] and references therein.

### 3.3. Electronics and Noise

**Figure 5:**
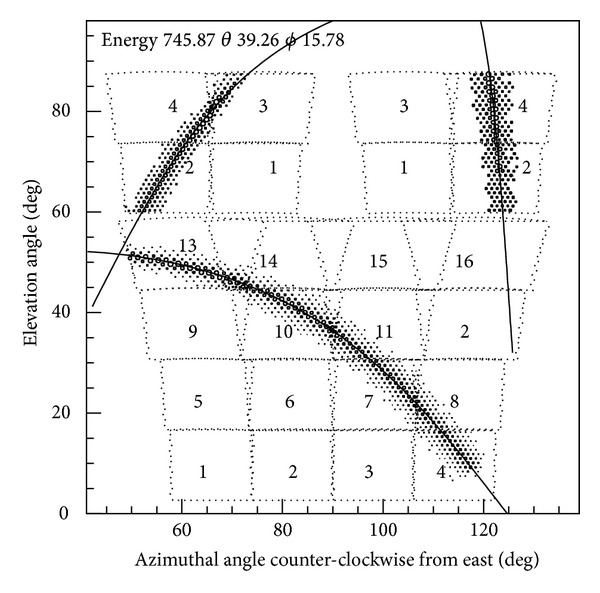
A typical air shower seen by the WFCTA fluorescence detectors. Each square marked with a number shows the FOV of each telescope. The open circles in the square represent triggered tubes and the size of each circle is proportional to logarithm of the number of photons. The solid curve crossing the circles represents the projection of a plane containing the shower axis and the detector. Energy (in PeV) and zenith and azimuth angle (in degrees) are displayed at the top.

All photons collected by one PMT are distributed in flash ADC bins according to their arrival time. Night sky background (NSB) photons with an average flux of 40 pe*μ*s^−1^m^−2^ [14] are randomly added to the waveform. The electronic noise with a mean of 1.2 FADC counts is also added to every 20 ns time window with fluctuations. The Trigger algorithm of the three levels as described in Section 2 is used in the simulation. An example of a detected CR event is shown in Figure 5.

## 4. Simulation Results

Using the simulation procedure and the optimized detector configuration described above, the detector aperture and event rate have been estimated.

### 4.1. Detector Aperture and Event Rate

**Figure 6:**
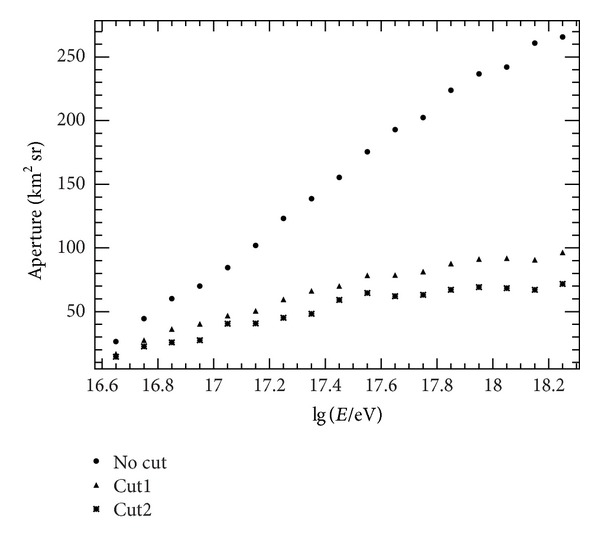
Detector aperture in different conditions. Solid circles are the aperture without any cut. Solid triangles are obtained using cut1 described in the text. Asterisks show the results obtained using cut2.

The detector triggered aperture has been estimated. This is displayed in Figure 6 with solid circles.

It can be seen in Figure 5 that the tower detector has two vertical edges that could cause an incomplete measurement of images, which may cause errors in estimates of energy and direction. To maintain event reconstruction quality, showers that touch the detector boundaries, with an angle between image center (weighted by signals) and mirror edge smaller than 4°, should be cut. To avoid Cherenkov light contamination, all tubes that have viewing angles smaller than 20° are removed and track lengths smaller than 10° are cut. Furthermore, at the WFCTA site located at a height of 4.3 km above sea-level, some showers with large *X*_max⁡_ will hit the ground before reaching the *X*_max⁡_. On the other hand, for some showers having small *X*_max⁡_, the location of maximum shower development will fall outside the upper edge of the tower array FOV. To guarantee the required resolution in the measurement of *X*_max⁡_, the showers' *X*_max⁡_ location must fall inside the detector FOV. There are about 18% of showers with *X*_max⁡_ falling outside the detector FOV. We denote these cut conditions by cut1, after applying cut1, the detector aperture is reduced as shown in Figure 6 by solid triangles.

**Figure 7:**
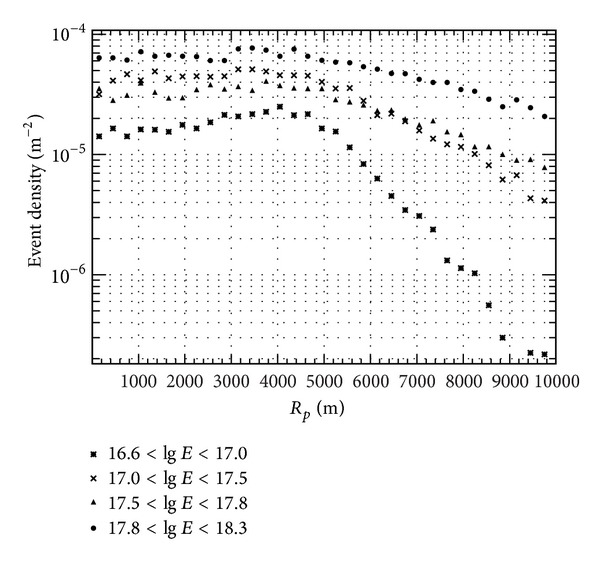
Event density distribution as a function of *R*_*p*_. Different points represent different energies.

**Figure 8:**
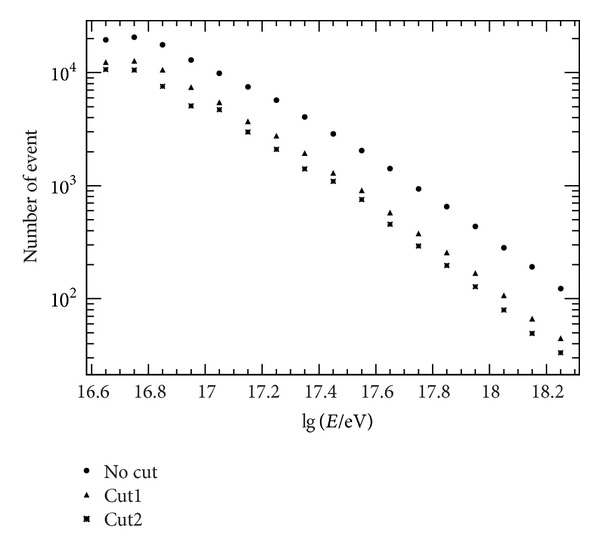
Event rate per year in three conditions, respectively.

In order to measure the structure of the cosmic ray energy spectrum around 10^17.5^ eV with minimum bias, a flat aperture as a function of energy is required. The triggered event density distributions as a function of *R*_*p*_ in different energy regions are shown in Figure 7. According to such an event density distribution, following geometric constraint based on *R*_*p*_ is applied. The events in the lg *E* intervals 16.6~17.0, 17.0~17.8, and 17.8~18.3 have *R*_*p*_ smaller than 5.5 km, 7.0 km, and 8.0 km, respectively. Cut1 together with geometric constraint is denoted cut2. The application of cut2 will yield a flat aperture as shown in Figure 6 by asterisks. The corresponding event rates are displayed in Figure 8. After all cuts, 14.3 k events with lg *E* > 17.0 and 162 events with lg *E* > 18.0 are obtained per year.

### 4.2. *X*_max⁡_ Distribution

**Figure 9:**
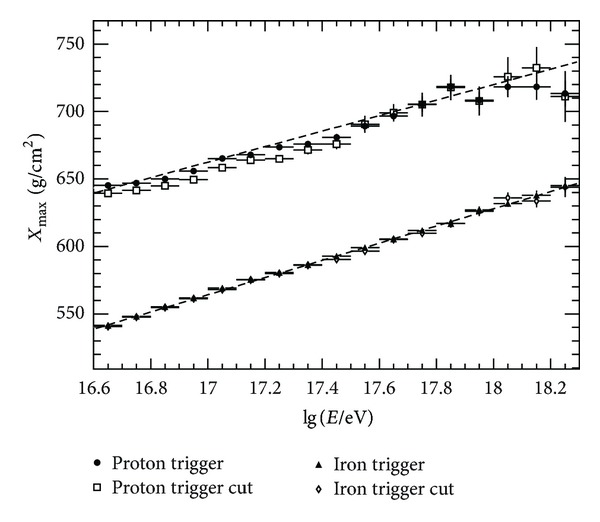
*X*_max⁡_ distribution as a function of energy. Two lines are for primary proton and iron. Solid points are triggered events without any cut. Opened points are for events after cut1 described in Section 4.1.

The *X*_max⁡_ distributions as a function of energy are displayed in Figure 9. The two point-curves are for primary proton and iron nuclei. The solid points are triggered events without any cut. The open points are for events after cut1 as described in Section 4.1. It indicates that the maximum detected bias is smaller than 10 g/cm^2^. Estimations of the *X*_max⁡_ resolution are ongoing.

## 5. Summary

The WFCTA fluorescence detector array simulation procedure has been completed. Based on the simulation tools, the detector array configuration optimization has been performed. To maintain data reconstruction quality, some cuts have been applied. In order to obtain the flat aperture needed for a precise study of the energy spectrum, further cuts have been applied. Detector apertures and event rates under three conditions have been evaluated. 14.3 k events with lg *E* > 17.0 and 162 events with lg *E* > 18.0 are left after all cuts. The *X*_max⁡_ distribution as a function of energy has been obtained. There is only a small detection bias both before and after event cuts. Therefore, it will be possible to carry out a detailed study of the CR energy spectrum and composition in the Sub-EeV range using such a fluorescence telescope array.
